# Eps homology domain endosomal transport proteins differentially localize to the neuromuscular junction

**DOI:** 10.1186/2044-5040-2-19

**Published:** 2012-09-13

**Authors:** Suzanne E Mate, Jack H Van Der Meulen, Priyanka Arya, Sohinee Bhattacharyya, Hamid Band, Eric P Hoffman

**Affiliations:** 1 Research Center for Genetic Medicine, Children’s National Medical Center, Washington, DC, USA; 2 Department of Integrative Systems Biology, George Washington School of Medicine, Washington, DC, USA; 3 Eppley Institute for Research in Cancer and Allied Diseases, UNMC-Eppley Cancer Center, University of Nebraska Medical Center, Omaha, NE, USA; 4 Department of Genetics, Cell Biology and Anatomy, College of Medicine, University of Nebraska Medical Center, Omaha, NE, USA; 5 Department of Pathology and Microbiology, College of Medicine, University of Nebraska Medical Center, Omaha, NE, USA

**Keywords:** Neuromuscular junction, Eps homology domain containing protein, Endosomal transport, Endosomal recycling, Bungarotoxin, Endplate, Synapse, Skeletal muscle

## Abstract

**Background:**

Recycling of endosomes is important for trafficking and maintenance of proteins at the neuromuscular junction (NMJ). We have previously shown high expression of the endocytic recycling regulator Eps15 homology domain-containing (EHD)1 proteinin the *Torpedo californica* electric organ, a model tissue for investigating a cholinergic synapse. In this study, we investigated the localization of EHD1 and its paralogs EHD2, EHD3, and EHD4 in mouse skeletal muscle, and assessed the morphological changes in EHD1^−/−^ NMJs.

**Methods:**

Localization of the candidate NMJ protein EHD1 was assessed by confocal microscopy analysis of whole-mount mouse skeletal muscle fibers after direct gene transfer and immunolabeling. The potential function of EHD1 was assessed by specific force measurement and α-bungarotoxin-based endplate morphology mapping in EHD1^−/−^ mouse skeletal muscle.

**Results:**

Endogenous EHD1 localized to primary synaptic clefts of murine NMJ, and this localization was confirmed by expression of recombinant green fluorescent protein labeled-EHD1 in murine skeletal muscle *in vivo.* EHD1^−/−^ mouse skeletal muscle had normal histology and NMJ morphology, and normal specific force generation during muscle contraction. The EHD 1–4 proteins showed differential localization in skeletal muscle: EHD2 to muscle vasculature, EHD3 to perisynaptic regions, and EHD4 to perinuclear regions and to primary synaptic clefts, but at lower levels than EHD1. Additionally, specific antibodies raised against mammalian EHD1-4 recognized proteins of the expected mass in the *T. californica* electric organ. Finally, we found that EHD4 expression was more abundant in EHD1^−/−^ mouse skeletal muscle than in wild-type skeletal muscle.

**Conclusion:**

EHD1 and EHD4 localize to the primary synaptic clefts of the NMJ. Lack of obvious defects in NMJ structure and muscle function in EHD1^−/−^ muscle may be due to functional compensation by other EHD paralogs.

## Background

The neuromuscular junction (NMJ) is a subcellular specialization of the myofiber plasma membrane, with nuclear domains directing synaptic gene expression. Our long-term goal is to provide a more complete molecular model of the NMJ. We previously identified concordance of the mammalian NMJ protein components with those of the *Torpedo californica* electric organ, describing the developmental origins of the organ and its extreme development into an amplified cholinergic synapse relative to skeletal muscle, to support its use as a model NMJ for hypothesis generation [1]. We identified several high-abundance proteins including Eps 15 homology domain-containing 1 (EHD1; Swiss-Prot:Q5E9R3), adducin gamma (ADD3; Swiss-Prot:Q9UEY8), laminin receptor protein 1 (LamR1; Swiss-Prot:Q803F6), chromosome 1 open reading frame 123 (C1orf123; Swiss-Prot: Q9NWV4), transgelin-3 (TAGL3; Swiss-Prot: P37805), and transforming growth factor-β-induced (TGFBI; Swiss-Prot:Q15582), which may play a role in synapse structure and maintenance. This approach of using the proteomic profile of an amplified model synapse should expedite candidate NMJ protein identification and characterization and thus help inconstructing a more complete NMJ paradigm.

In the current study, EHD1 was examined because of the high number of unique peptides (n = 20) identified in the electric organ proteome relative to mouse skeletal muscle (n = 0), and its high spectral cross-correlation value (140). In addition, EHD1 was investigated as a peripheral membrane protein that functions in clathrin-independent endocytosis and recycling of receptors at the membrane through the tubular endosomal recycling compartment (ERC) [1,2].

The EHD family of proteins (EHD1 to EHD4) contain an EH domain that facilitates interactions with proteins encoding asparagine-proline-phenylalanine (NPF) motifs, which form complexes that regulate endocytic trafficking [3,4]. The current functional paradigm for this group of proteins is that EHD3 and EHD4 assist in the transport of proteins from the early endosome (EE) into the ERC whereas EHD1 and EHD2 assist in the cargo exit from the ERC to the plasma membrane [4]. In addition to the C-terminal EH domain that EHD proteins share with many proteins of the endocytic machinery, EHD family proteins share a central coiled-coil and an N-terminal phosphate binding loop (P-loop) [3,5]. These proteins are products of gene duplication, are encoded on separate chromosomes, and have differential expression profiles in various tissues [3,4,6-8]. In adult tissues, EHD1 is expressed in germ cells, adipocytes, the eye (retina, rods and cones outer nuclear layer, internal nuclear layer, and ganglion cell layer), the basal membrane of the endometrium and uterine muscle cells, granulosa cells after ovulation, skeletal muscle, kidney, and spermatocytes, but it has not been found in spleen, liver, or brain [3]. The EHD1 protein has been studied in multiple cultured cells, whole-tissue extracts, and the testis; however, its subcellular localization in normal tissues has not been characterized.

Several proteins known to serve as components of presynaptic and postsynaptic membranes contain NPF domains, suggesting their potential interaction with the EH domain of EHD1 and/or other family members. At the presynaptic membrane these include stoned (stnB), synaptosomal-associated protein (Snap)29, secretory carrier membrane proteins (SCAMP)1 and SCAMP5, and syndapin I (also known as Pacsin I). Each of these proteins functions as part of the syanaptosome that regulates vesicle transport and neurotransmitter release across the NMJ [9-14]. Interestingly, the EH domain of EHD1 binds snapin, a soluble *N*-ethylmaleimide-sensitive factor attachment protein receptors (SNARE)-associated protein that does not contain an NPF motif, effectively blocking exocytosis of synaptic vesicle [15]. Furthermore, neuronal-glial cell adhesion molecule (NgCAM) trafficking is dependent on EHD1 [16]. Overall, EHD proteins are thought to be an important component of the presynaptic synaptosome.

In the postsynaptic membrane, ankyrins are known to stabilize membrane and membrane-associated proteins at the NMJ [17]. Expression of EHD1-4 proteins was increased in ankyrin-B^−/−^ cardiomyocytes [18]. Functional studies in HeLa cells showed that EHD1 regulates the expression of β1 integrin via a clathrin-independent mechanism and Arf6 and Rab family proteins [8]. β1 integrin is a key extracellular-matrix (ECM) receptor that facilitates interactions with the ECM at focal adhesions but is also a key mediator of downstream signaling pathways important for cell survival, growth, environmental sensing, and cellular movement [19,20]. Furthermore, β1 integrin has high expression in skeletal muscle and forms a dimer with α7 integrin at the NMJ, where it synergistically interacts with agrin and laminins 1 and 2/4 to promote acetylcholine receptor (AChR) clustering during maturation of muscle and AChR stability throughout life [21], supporting the potential role of EHD1 at the NMJ.

Maintenance and signaling of postsynaptic receptors is intimately linked to their turnover and trafficking, suggesting a possible role for EHD1 and its paralogs. Both ErbB receptor tyrosine kinases and muscle-specific tyrosine-protein kinase receptors (MuSK) are thought to signal after endocytosis into vesicles containing the downstream proteins that initiate synaptic gene transcription, reorganization of the cytoskeletal network, and clustering of AChRs or gene transcription (the so-called signaling endosome hypothesis). Earlier studies showed that ligand-induced endocytosis of MuSK occurs via a clathrin-independent but lipid-raft-dependent pathway [22,23]. By contrast, ErbB receptor tyrosine kinases are internalized through the clathrin-mediated endocytic pathway upon neuregulin binding to activate AChR expression [24]. Interestingly, the NPF-domain containing protein phosphatidylinositol-binding clathrin assembly protein (CALM) functions in AP-2-dependent clathrin-mediated endocytosis [25], potentially by intersecting with EHD proteins. Furthermore, tyrosine phosphorylation is crucial for recycling of endocytosed AChR to the synaptic crests; inhibitors of tyrosine phosphorylation cause AChR to become trapped or located in perisynaptic regions [26].

Given the role of EHD1 in the transport of ligand-bound receptors, and given the importance of ligand-bound receptors such as MuSK and ErbB in postsynaptic stabilization and gene expression, we hypothesized that EHD proteins might play an important role in AChR clustering and postsynaptic membrane architecture. We report the localization and function of a previously unknown NMJ protein, EHD1.

## Methods

### Animal husbandry and care

All procedures performed on mice were approved by the Children’s National Medical Center Institutional Animal Care and Use Committee. Three male EHD1^−/−^ (3, 6, and 15 months old) and three C129/C57BL/6 J (4, 6, and 15 months old) mixed background mice (kindly provided by Dr Band, University of Nebraska Medical Center) were used [4,7]. C57BL/6 J/J mice (The Jackson Laboratory, Bar Harbor, MN, USA) were used for recombinant protein expression and localization studies. We used this small number of mice because of the difficulty in obtaining viable EHD1^−/−^ mice that survived beyond 10 postnatal days; EHD1^−/−^ mice survive at lower than mendelian levels because of their low mean body size, malocclusion-induced malnutrition, and other unknown causes [7].

### DNA amplification and purification of candidate genes

A number of expression vector clones (pReceiver; OmicsLink Expression Clones; GeneCopoeia, Rockville, MD, USA), containing the cDNA insert for NMJ proteins with both C-terminal (M03) and N-terminal (M29) green fluorescent protein (GFP) sequence tag were used (GFP control (EX-EGFP-M03), rapsyn (EX-Mm04872-M03/M29), EHD1 (EX-Mm02286-M03/M29)). Constructs were used to transform *Escherichia coli* (GCI-5α™ Chemically Competent *E. coli* Gene Copoeia™) in accordance with the company’s guidelines (*Transformation Protocol for cDNA Clones from Filter Paper Discs*). The transformed *E. coli* were grown overnight at 37°C with continuous shaking at 250 rpm in Luria broth with ampicillin 100 μg/ml (Sigma, St. Louis, MO, USA) added for selection of transformed cells. Plasmids were purified using a commercial kit (PureLink™ HiPure Plasmid Midiprep Kit; Invitrogen, Carlsbad, CA, USA) in accordance with the manufacturer’s directions. All purified plasmids were reconstituted in sterile phosphate-buffered saline (PBS) and measured on a spectrophotometer (ND 1000; Thermo Scientific, Wilmington, DE, USA) for approximating purity and concentration (OD A260/A280 >1.8). Purified plasmids were stored at 4°C or at −20°C depending on the time of use.

### Intramuscular injection of naked DNA into mouse tibialis anterior muscle

C57Bl/6 J mice (6 to 10 weeks old were anesthetized with 1–3% isofluorane/O_2_. Their hind legs were shaved and then wiped with 70% isopropyl alcohol. Five injections (5 μl each) of a plasmid suspension in sterile PBS (1 μg/μl) were given intramuscularly into the tibialis anterior (TA) muscle using a 26 gauge needle (RN NDL 26/2′/2 s) and a 25 μl syringe (802RN 22 s/2’/2) (both Hamilton Reno, NV, USA). At 3 or 14 days after the injection, the mice were killed using carbon dioxide and cervical dislocation.

### Immunofluorescent microscopy of whole-mount isolated muscle fibers for Green fluorescent protein localization

The mouse TA muscle was fixed *in situ* with 2% paraformaldehyde in PBS at the time of dissection, and stored overnight in 10% sucrose/PBS at 4°C as previously described [27]. The TA muscle was manually teased apart into small fiber bundles and incubated in blocking and permeabilization buffer (PBS containing 0.5% TritonX-100, 0.1% Tween 20, 2% bovine serum albumin (BSA) and 20% horse serum) overnight at 4°C. Fibers were incubated with anti-GFP rabbit IgG fraction Alexa Fluor 555 conjugate (Invitrogen) 1:400 dilution in antibody buffer (PBS containing 0.2% TritonX-100, 0.1% Tween 20, 2% BSA in PBS) for 2 hours with gentle mixing. Fibers were separated by centrifugation at 4000 *g* for 1 minute, then washed with 0.1% Tween-20 in PBS, repeating this cycle three times. Fibers were counterstained with α-bungarotoxin (BTX) Alexa Fluor 488 conjugate (Invitrogen) 1:3000 in PBS for 10 min to label NMJs, then washed three times, counterstained with 4^′^,6-diamidino-2-phenylindole (DAPI, dilactate; Invitrogen) 1:5000 in double-distilled H_2_O to label nuclei, and finally washed three more times. Fibers were left in wash buffer overnight at 4°C with gentle mixing, then mounted (Fluoromount-G; Southern Biotech, Birmingham, AL, USA). Confocal laser-scanning microscopy was performed using a laser-scanning microscope (LSM 510) coupled to microscopy software (Zen LE) (both Carl Zeiss, Jena, Germany). Qualitative analysis of recombinant protein localization was assessed relative to BTX labeling of NMJs.

### Immunofluorescent microscopy of whole-mount isolated muscle fibers for Eps15 homology domain-containing protein localization and neuromuscular junction morphological mapping

Fibers from EHD1^−/−^ and C129/C57BL/6 J mice were prepared for confocal microscopy as described above, except for the use of the following EHD antibodies, previously generated and described [4]. Fibers were labeled with 1:200 rabbit anti-EHD1, EHD2, EHD3, and EHD4 antibodies and 1:500 Alexa Fluor 555 F(ab')2 fragment of goat anti-rabbit IgG (H + L; Invitrogen). Peripheral nerves were labeled with 1:500 neuronal class III β-tubulin monoclonal antibody (TUJ1; Covance, Princeton, NJ, USA) and 1:250 Alexa Fluor 568 goat anti-mouse IgG (H + L) secondary antibody (Invitrogen). Fibers that were only counterstained with BTX Alexa Fluor 555 conjugate (Invitrogen) 1:3000 in PBS and with DAPI (Invitrogen) 1:5000 in double-distilled H_2_O were not incubated in the blocking and permeablization buffer, but were placed in PBS for immediate counterstaining and mounting as described above. Qualitative analysis of immunolabeled protein localization was assessed relative to BTX labeling of NMJs.

### Western blotting detection of Eps15 homology domain-containing protein proteins to investigate conservation across species

The gastrocnemius muscles were dissected and flash frozen in liquid nitrogen. Muscle was ground in a mortar and pestle set in a dry ice bath, cooled with liquid nitrogen, and homogenized using a hand homogenizer in lysis buffer (0.25 mol/l sucrose, 20 mmol/l Tris pH 8.0, 25 mmol/l KCl, 5 mmol/l MgCl_2_, and protease inhibitor (Mini Complete Protease Inhibitor; Roche Diagnostics, Basel, Switzerland). The homogenates were left on ice for 30 minutes, and were then sonicated on ice for 30 pulses (50% duty cycle, pulsed-hold, output control limit 3; Sonifier Cell Distributer 350; Branson Scientific, Danbury, CT USA), followed by an additional 30 minutes on ice, after which homogenates were separated by centrifugation at 10,000 *g* for 15 minutes at 4°C. for each homogenate, the supernatant was collected and the pellet was suspended in EBC buffer (50 mmol/l Tris–HCl pH 8.0, 120 mmol/l NaCl, 1% Triton-X 100, and protease inhibitor (as before; Roche). All lysates were desalted by passing the sample through a BioSpin6 micro column before protein quantification (DC Protein Assay; BioRad, Hercules, CA, USA). The protein extracts were stored at 80°C until electrophoresis. For each lysates, 20 μg of protein was loaded onto a one-dimensional SDS-PAGE gel (Novex NuPage 4–12% Bis-Tris MiniGel Systems; Invitrogen) in 2-(N morpholino) ethanesulfonic acid (MES) buffer (Invitrogen) at room temperature or 3-(N-morpholino)propanesulfonic acid (MOPS)buffer at 4°C, then transferred to nitrocellulose membrane (Amersham Hybond ECL; GE Healthcare, Piscataway, NJ, USA) at a constant current of 0.02 amps overnight at 4°C in transfer buffer (NuPage Transfer Buffer (Invitrogen, Carlsbad, CA, USA) in accordance with manufacturer’s directions. Blots were rinsed in TBS-T buffer (Tris–HCl pH 7.5 (Invitrogen, Carlsbad, CA) plus 0.1% Tween20 (Sigma)), blocked in 5% blotting grade milk (BioRad) in TBS-T for 1 hour at room temperature, then probed with rabbit anti-EHD1-4 antibodies overnight at 4°C/ EHD1 and EHD4 were co blotted because of the cross reactivity of the EHD1 antibody. Blots were rinsed several times in TBS-T buffer, probed with 1:2000 goat anti-rabbit IgG H + L HRP secondary antibody (Invitrogen) for 1 hour at room temperature, rinsed again, developed using a commercial kit (Amersham ECL Kit), and exposed to film (Amersham HyperFilm ECL) (both GE Healthcare). Blots were then stripped (0.35 ml of 100 mmol/l 2-mercaptoethanol and 2% SDS in 10 ml of 62.5 mmol/l Tris–HCl pH 6.8, 3.125 ml of 62.5 mmol/l Tris–HCl pH 6.8, made up to 50 ml with distilled water) for 1 hour at 50°C, rinsed, blocked, and reprobed for GAPDH (14 C10; rabbit monoclonal antibody diluted 1:4000 in TBS-T with 3% BSA in TBST; #2118, Cell Signaling, Danvers, MA, USA) using the same secondary antibody as stated above. Soluble and insoluble extracts of *T. californica* were resolved by SDS-PAGE and transferred to nitrocellulose membrane to blot for EHD1-4 proteins as described above,except that in this case we used MOPS running buffer, 1:20,000 goat anti-rabbit IgG H + L HRP secondary antibody, and a different substrate (SuperSignal West Pico Chemiluminescent Substrate‘ Thermo Scientific, Rockford, IL. USA). We performed a qualitative analysis of EHD1-4 protein expression in wild-type (WT) and EHD1^−/−^ mouse skeletal muscle and *T. californica* electric organ fractions.

### Muscle dissection for *in vitro* force measurements

Mice were anesthetized with intraperitoneal injections of ketamine 70 mg/kg and xylazine 7 mg/kg. Supplemental doses were administered as needed to maintain adequate levels of anesthesia throughout the dissection of the muscles. Experiments were conducted on the Extensor digitorum longus (EDL) and soleus muscle of the right hind limb from control (n = 3) and EHD1 null (n = 3) mice. The EDL muscle was isolated and 6–0 silk sutures were tied securely to the distal and proximal tendon. The muscle was then removed from the mouse and placed in a vertical bath containing buffered mammalian Ringer solution (37 mmol/l NaCl, 24 mmol/l NaHCO_3_, 11 mmol/l glucose, 5 mmol/l KCl, 2 mmol/l CaCl_2_, 1 mmol/l MgSO_4_, 1 mmol/l NaH_2_PO_4_, and 0.025 mmol/l turbocurarine chloride, pH 7.4 at 25°C, and bubbled with 95% O2/5% CO_2_. The distal tendon of the muscle was tied securely to the lever arm of a servomotor/force transducer (model 305B; Aurora Scientific, Richmond Hill, ON, Canada) and the proximal tendon to a fixed clamp. After performing a protocol of force contractions, the same dissection procedure was used for the soleus muscle. After removal of the soleus muscle, animals were killed with an overdose of CO_2_.

### Measurement of contractile properties *in vitro*

Muscles were stimulated between two platinum electrodes. Using supramaximal stimulation of the muscle with single 0.2-ms square stimulation pulses, muscle length was adjusted to the length (Lo) that resulted in maximal twitch force. With the EDL muscle held at Lo^′^, the muscle was stimulated for 300 ms with increasing stimulation frequencies of 30, 50, 80, 100, 120, 150, 180, 200, 220 and 250 Hz to establish the plateau of force generation (maximum isometric tetanic force, Po). Each stimulus was separated by 2 minutes of rest. A similar protocol was applied to the soleus muscle, but the stimulation frequencies were 30, 50, 80 and 100 Hz and the stimulus duration was 1000 ms. The muscle length was then measured with calipers, and after removal of the muscle from the bath, the mass of the muscle was determined. The optimum fiber length (Lf) was calculated by multiplying Lo by a previously determined Lf/Lo ratio of 0.45 for the EDL muscle and by 0.71 for the soleus muscle [28]. Total muscle fiber cross-sectional area was determined by dividing the wet mass by the product of Lf and the density of mammalian skeletal muscle (1.06 mg/mm^3^). Maximum isometric specific force (sPo) was determined by dividing Po by the total muscle fiber cross-sectional area.

### Statistical analysis

Student’s *t*-test was used to compare the sPo means of WT and EHD1^−/−^ skeletal muscle, with *P* < 0.05 considered significant we performed stats in Excell with the Data Analysis add-in. We performed F-tests followed by t-tests, which are described in the Results section with the data.

## Results

### Eps15 homology domain-containing 1 protein localizes to the primary synaptic cleft in skeletal muscle

Our proteomic data identified EHD1 as an abundant protein in the *T. californica* electric organ [1], suggesting that mammalian EHD1 might be an uncharacterized candidate neuromuscular junction protein. To investigate the expression and localization profile of EHD1, the complete murine cDNA was cloned into M03 and M29 vectors (GeneCopoeia) for expression of EHD1 with N- or C-terminal GFP tags, respectively, under control of a cytomegalovirus (CMV) immediate-early promoter (EX-Mm02286-M03/M29). For each recombinant protein expressed, 25 g of purified DNA in PBS was injected directly into the TA muscle of four WTC57BL/6 J)mice as fiveinjections of 5 μleach to transfect a subset of myofibers. Skeletal muscle autofluoresces near the same wavelength as GFP, thus to reduce this innate background signal, anti-GFP Alexa 555 was used to detect recombinant protein expression. Morphological mapping of NMJs was carried out by labeling AChRs at the postsynaptic membrane with BTX Alexa 488 conjugate. The high density of these receptors generated an intense signal that delineates the primary synaptic cleft of mature endplates. We assessed localization of EHD1 relative to this NMJ marker using confocal microscopy.

**Figure 1:**
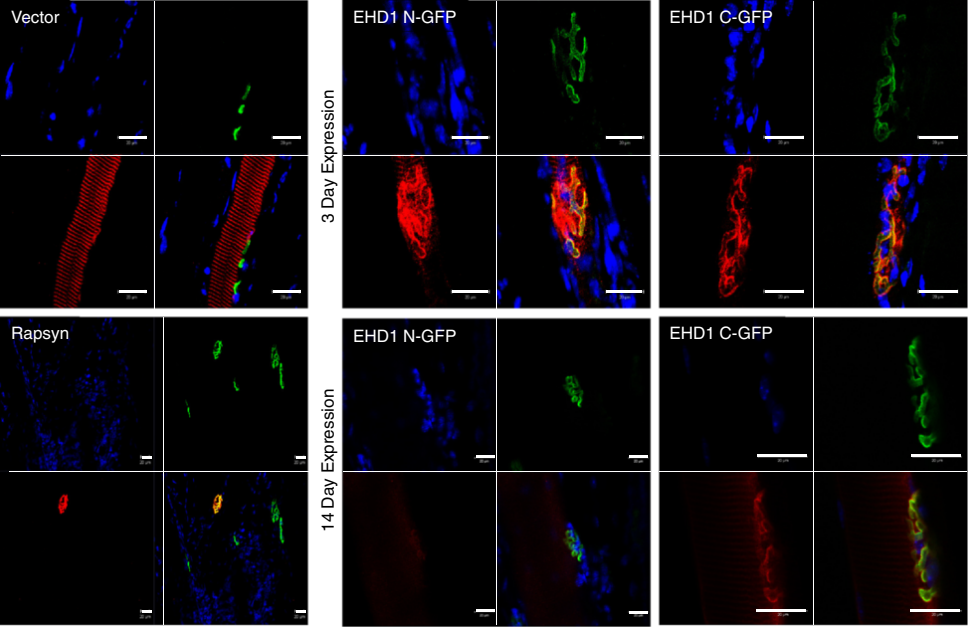
**Recombinant green fluorescent protein (GFP)-tagged Eps15 homology domain-containing (EHD) protein localizes to the neuromuscular junction (NMJ).** Control and EHD1 proteins were expressed for 3 or 14 days in 6- to 10-week-old C57Bl/6 J mouse tibialis anterior (TA) skeletal muscle as C-terminal and N-terminal GFP recombinant proteins. Four mice were injected for each recombinant protein expressed. The TA muscle was fixed *in situ* and teased into small bundles for immunofluorescence and visualization via confocal microscopy, referenced to α-bungarotoxin (BTX) labeling of AChRs at the NMJ. Expression of GFP (vector) was seen as a diffuse signal throughout the myofiber and did not concentrate at the NMJ (n = 7). Rapsyn-GFP expression localized exclusively to the NMJ of transfected fibers (n = 2). Both transfected and non-transfected fibers are shown in the bottom left panel. N- and C-terminal GFP recombinant EHD1 proteins localized to the NMJ and to the primary synaptic cleft with BTX after 3 or 14 days of transfection (n = 24). Each image consists of separate blue (DAPI), green (BTX Alexa Fluor 488 conjugate), and red (anti-GFP Alexa Fluor 555 conjugate) channels in addition to a merged image. Scale bars: 20 μm. The larger reference bar was added in powerpoint to make the 20 μm bar visible in the images. It reflects the 20 μm scale bar that was added within the Zen software upon image acquisition.

To demonstrate the properties of CMV promoter-driven recombinant protein expression in mouse skeletal muscle with subsequent localization analysis by confocal microscopy, GFP and rapsyn were expressed *in vivo* for 3 days to serve as controls. Expression of GFP, a non-muscle protein, was seen as a diffuse and non-specific distribution in the sarcoplasm, with no detectable aggregation of signal at the NMJ (Figure 1). Rapsyn, a protein located exclusively at the NMJ, localized specifically to the synapse without any detectable signal in extrasynaptic regions (Figure 1). Together, these controls support the use of the CMV promoter-driven recombinant gene expression of candidates from direct DNA injection for localization analyses. EHD1 N-terminal and C-terminal GFP-tagged recombinants expressed for 3 or 14 days all showed localization at the synapse (Figure 1). Diffuse immunostaining of EHD1 was seen in other areas of the myofiber, although at a lower intensity than in the NMJ.

**Figure 2:**
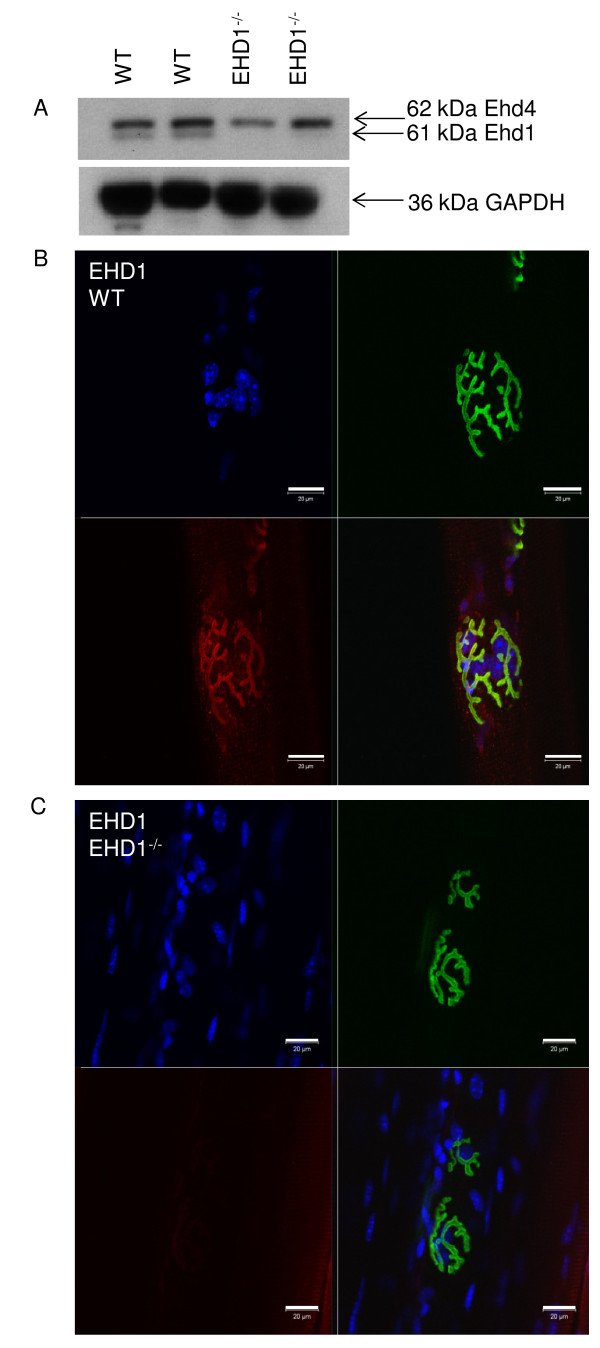
**Eps15 homology domain-containing (EHD)1 is expressed in mouse skeletal muscle and localizes to the primary synaptic clefts of neuromuscular junctions. (A)** Western blot of two WT and two EHD1^−/−^ skeletal muscle lysates probed with peptide-specific antibodies against EHD1 and EHD4 showedexpression in skeletal muscle and verified the loss of EHD1 in null mice. Glyceraldehyde 3-phosphate dehydrogenase (GAPDH) served as a loading control. **(B)**The tibialis anterior (TA) skeletal muscles of three wild-type (WT) and three EHD1^−/−^ mice were teased into small bundles for immunofluorescence and visualization via confocal microscopy. α-bungarotoxin (BTX) labeling of AChRs denote NMJs in all images. EHD1 localized to the NMJ and delineated the primary synaptic cleft similar to BTX labeling. **(C)** Because the EHD1 antibody crossreacts with EHD4, imparting low background signal, EHD1^−/−^ fibers were labeled with anti-EHD1 to show the specificity of the localization. Blue, DAPI; green,BTXAlexa Fluor 488 conjugate; red, anti-green fluorescent protein Alexa Fluor 555 conjugate. Scale bars: 20 μm. A larger reference bar was added above the scale bars.

To confirm and extend the EHD1-GFP fusion localization data, TA muscles were fixed *in situ* and teased apart into small myofiber bundles for immunofluorescence and confocal microscopy analysis using antibodies generated against EHD1 peptides. Characterization of EHD1 and EHD4 antibodies identified crossreactivity of EHD1 antibody with EHD4 protein, but not the reverse [4]. Western blotting of skeletal muscle lysates with EHD1 and EHD4 antibodies showed that each antibody was able to identify these two paralogs and identify their expression in skeletal muscle (Figure 2A). Myofiber bundles were isolated from the TA muscle of three 129/C57 BL6 mixed background (WT) and three EHD1^−/−^ mice for immunolocalization analysis by confocal microscopy [7]. Myofibers were counterstained with α-BTXAlexa 488 conjugate to denote the primary synaptic cleft of NMJs by labeling the AChRs, and with DAPI to stain nuclei. WT myofibers showed strong colocalization of EHD1 with α-BTXat the NMJ, and this signal was lost in the EHD1^−/−^ NMJs (Figure 2B,C). This confirmed the fusion protein data showing preferential localization of EHD1 at the myofiber NMJ.

### Eps15 homology domain-containingproteins 1–4 show differential localization in skeletal muscle

**Figure 3:**
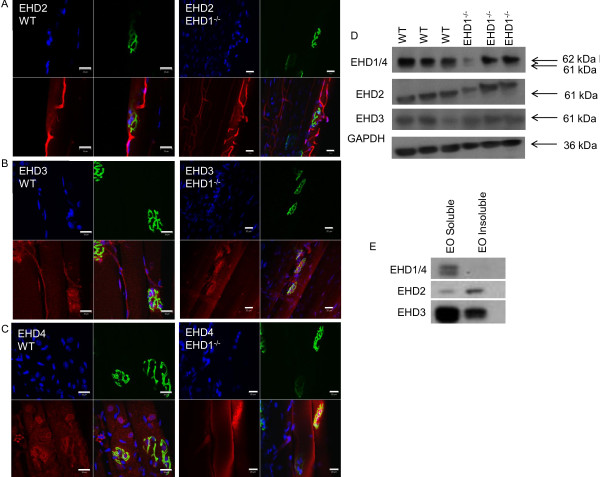
**Eps15 homology domain-containing (EHD) 1–4 proteins show differential localization in skeletal muscle.** The tibialis anterior (TA) skeletal muscles of three wild-type (WT) and three EHD1^−/−^ mice were teased into small bundles for immunofluorescence and visualization via confocal microscopy. α-bungarotoxin (BTX) labeling of AChRs denote neuromuscular junctions (NMJs) in all images. EHD2, 3, and 4 are expressed in WT and EHD1^−/−^ mice. Panel **(A)** EHD2 localized to skeletal muscle vasculature endothelium. **(B)** EHD3 localized to synaptic regions adjacent to theBTX labeling and in the vasculature, but to a lesser extent than EHD2. **(C)** EHD4 localized to the synaptic cleft with BTX in addition to adjacent extrasynaptic regions and perinuclear regions. **(D)** EHD 1–4 expression in skeletal muscle lysates was analyzed by western blotting. **(E)** EHD1-4 antibodies also recognized proteins of similar mass in *Torpedo californica* electric organ lysates. Blue, DAPI; green, BTX Alexa Fluor 488 conjugate; red, anti-rabbit Alexa Fluor 555 conjugate.Scale bars: 20 μm. A larger reference bar was added above the scale bars.

The *T. californica* proteomic data identified peptides that were shared between EHD paralogs and peptides unique to EHD1 [1]. We therefore extended the EHD1 localization studies in WT and EHD1^−/−^ muscle to EHD2, EHD3, and EHD4 using anti-peptide antibodies specific for their corresponding paralog [4]. Using the same isolated myofiber method for immunostaining, the TA muscle from three mice per group was analyzed. EHD2 signal was consistent with expression in muscle vasculature, suggesting localization in endothelial cells (Figure 3A). There was no evidence of immunostaining for EHD2 in the NMJ. The EHD3 signal was concentrated at the postsynaptic membrane but did not delineate the primary synaptic cleft as EHD1 did; instead, EHD3 localized to perisynaptic areas, the area adjacent to BTX-labeled NMJs, and to the vasculature, although to a lesser extent than EHD2 (Figure 3B). EHD4 immunolabeling delineated the striations and perinuclear regions to a greater extent than other family members, but had weaker staining of the primary synaptic cleft at lower levels than EHD1 (Figure 3C). Antibody labeling of EHD2 to EHD4 proteins in EHD1^−/−^ fibers showd similar staining patterns to those found in WT fibers (Figure 3).

Expression of *EHD1* gene transcripts in WT and EHD1^−/−^ skeletal muscle was confirmed by reverse transcriptase PCR (data not shown), verifying the lack of EHD1expression in EHD1^−/−^ mouse skeletal muscle [4]. Western blotting further validated the expression of EHD1 to EHD4 proteins in WT skeletal muscle and of all but EHD1 in EHD1^−/−^skeletal muscle (Figure 3D). Furthermore, western blotting using the same anti-peptide antibodies showed crossreactivity with the corresponding proteins in the *T. californica* electric organ with similar molecular weights to those of mammalian EHD proteins, suggesting a high degree of cross-species conservation (Figure 3E). With the exception of the two peptides that are unique to EHD1, all other peptides found in the electric organ proteome that were mapped to EHD1 were shared by EHD3.

### Eps15 homology domain-containing (EHD)4 shows increased expression and enrichment at the neuromuscular junction in EHD1^−/−^ myofibers

**Figure 4:**
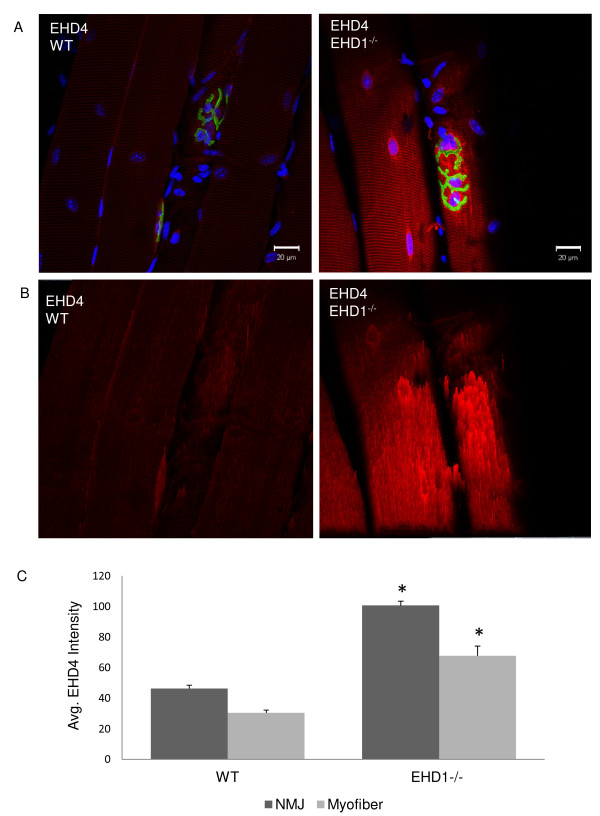
**Eps15 homology domain-containing (EHD)4 expression is increased in EHD1**^**−/−**^**muscle.** The tibialis anterior (TA) skeletal muscle of three wild-type (WT) and three EHD1^−/−^ mice were teased into small bundles for immunofluorescence labeling with anti-EHD4 antibody and z-stacks were acquired to assess signal throughout the entire myofiber diameter/thickness via confocal microscopy. α-bungarotoxin (BTX) labeling of AChRs denote NMJs. EHD4 signal intensities were compared in myofibers (n = 5) and at neuromuscular junctions (NMJs) (n = 10) from WT and EHD1^−/−^ mice (n = 3 per group) using identical acquisition parameters. **(A)** The z-section with the highest signal for EHD4 (red channel). **(B)** Intensity projections of the EHD4 signal from representative NMJs in (A). Blue, DAPI; green, BTX Alexa Fluor 488 conjugate; red, anti-rabbit Alexa Fluor 555 conjugate.Scale bars: 20 μm. A larger reference bar was added above the scale bars. **(C)** The average intensity of EHD4 expressed in WT and EHD1^−/−^ mice. The bars represent the average vector measurements of the intensity with standard error from myofibers and NMJs in each group. Statistical significance was measured by Student’s *t*-test (*P* < 0.05).

The signal intensity of EHD4 in EHD1^−/−^ mouse myofibers appeared to be greater than that in WT myofibers. Confocal z-stacks of WT and EHD1^−/−^ myofibers, dissected from three mice per group labeled with EHD4 antibody, were acquired under identical parameters (40× objective, 1 μm z-section interval, mean of four images, 1 AU pinhole (blue 9.9; green4.7; red 13.4), 720 gain on the blue channel, 725 gain on the green channel, and 531 gain on the red channel) to assess signal intensity levels. Representative WT and EHD1^−/−^ NMJs in EHD4 labeled myofibers (Figure 4) show the z-slice with greatest signal intensity was on the red channel. Intensity projections of the z-slice (Figure 4A) reinforced the abundant signal of EHD4 in EHD1^−/−^ myofibers, showing the previously described localization at the NMJ and at perinuclear regions in both WT and EHD1^−/−^ skeletal muscle (Figure 4B). However, the amplification of EHD4 expression in EHD1^−/−^ muscle was enriched throughout the length of the myofibers. Quantification of signal intensities throughout the entire myofiber, not specifically localized to the NMJ, were estimated using vector intensity measurements applied by the Zen software. Measurements were performed on representative fibers (n = 5), analyzed per animal (n = 3) per group (n = 2). The mean intensity per group was assessed for significance using a one-tailed Student’s *t*-test assuming unequal variance, which was determined using the *F*-test, with *P* < 0.05. EHD4 signal intensity levels were significantly greater in EHD1^−/−^ myofibers (Figure 4C). Furthermore, we assessed the intensity of EHD4 at NMJs in representative fibers (n = 10) from each mouse (n = 3) per group (n = 2) using the same method. EHD4 signal intensity levels were also significantly greater at the NMJ of EHD1^−/−^ myofibers (one-tailed Student’s *t*-test assuming unequal variance, which was determined using the *F*-test, with *P* < 0.05; Figure 4C).

### Neuromuscular junction morphology is generally maintained in Eps15 homology domain-containing (EHD)1^−/−^ skeletal muscle

**Figure 5:**
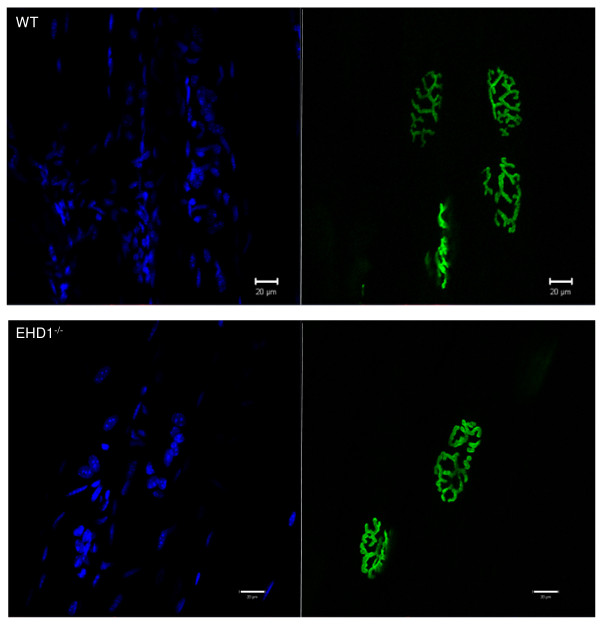
**Neuromuscular junctions (NMJs) in Eps15 homology domain-containing (EHD)1**^**−/−**^**mice show no morphological defects in synaptic endplate structure.** (A) The tibialis anterior (TA) skeletal muscle fibers from three EHD1^−/−^ and three wild-type (WT) mice were labeled with α-bungarotoxin (BTX) Alexa Fluor 555 or 488 conjugate for morphological mapping of endplates using confocal microscopy. NMJs were seen in both WT (n = 260) and in EHD1^−/−^ (n = 275) fibers. Blue, DAPI; green, BTX Alexa Fluor 488 conjugate; red, anti-rabbit Alexa Fluor 555 conjugate. Scale bars: 20 μm. A larger reference bar was added above the scale bars.

The high specificity of α-BTX for AChR at the NMJ synaptic crests permitted us to study the synaptic endplate structure with fluorescence confocal microscopy. Morphological mapping of NMJs in TA skeletal muscle fibers of 3-, 6-, and 15-month-old EHD1^−/−^ mice showed that most endplates had morphology similar to the NMJs of 4-, 6-, and 15-month-old WT mice (Figure 5). For morphological assessment of endplate structure, 260 WT and 275 EHD1^−/−^ NMJs were analyzed. Furthermore, NMJs in WT and EHD1^−/−^ mice did not differ in size; the length of 68 NMJs per group had equal variance, determined using the *F*-test, and no significant difference in mean size was seen (Student’s *t*-test, *P* < 0.05; data not shown).

**Figure 6:**
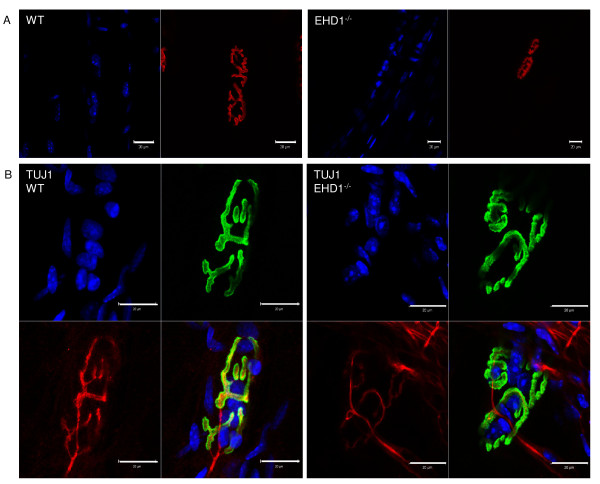
**Neuromuscular junctions (NMJs) of Eps15 homology domain-containing (EHD)1**^**−/−**^**mice are innervated by a single motor axon.** Panel **(A)** Isolated tibialis anterior (TA) skeletal muscle fibers from three EHD1^−/−^ and three wild-type (WT) mice were labeled with (BTX) Alexa Fluor 555 conjugate for morphological mapping of endplates using confocal microscopy. Rare EHD1^−/−^endplates (n = 2) consisted of two discreet structures, with one being smaller and less developed than the other. **(B)** Isolated TA skeletal muscle fibers from three EHD1^−/−^ and three WT mice were immunolabeled with neuronal class III β-tubulin monoclonal antibody (TUJ1) antibody, a neuronal marker, and visualized using confocal microscopy. WT (n = 13) and EHD1^−/−^ (n = 12) endplates with partitioning were seen. EHD1^−/−^ endplates were singly innervated, even when partitioned. Blue, DAPI; green, α-BTXAlexa Fluor 488 conjugate; red, anti-mouse Alexa Fluor 568 conjugate. Scale bars: 20 μm. A larger reference bar was added above the scale bars.

Although rare, a subset of NMJs displayed partitioned synaptic structure in EHD1^−/−^ skeletal muscle. The characteristic ‘pretzel-like’ morphology of the WT endplates was discontinuous, forming two discrete endplates, with regions showing little architecture (Figure 6A). This morphology cannot be explained by age-dependent endplate fragmentation, because the primary clefts remained continuous and were not separated into the small ‘islands’ typically seen at 18 months of age, when significant number of fibers show age-specific alterations [29]. Furthermore, we did not find near these partitioned endplates any centrally located nuclei that would indicate an injury-induced regenerated endplate from that formed during development. This may indicate incomplete pruning, axon sprouting from a single motor neuron that has two distinct terminal endings, or altered AChR trafficking or clustering beneath these endplates.

To study the number of nerve termini innervating NMJs, motor axons were immunolabeled for confocal microscopy analysis using an antibody specific for the neurofilament TUJ1. The TA muscles from three mice per group were used for the analysis of partitioned endplates; 13 partitioned NMJs were seen in WT muscle and 12 in EHD1^−/−^ muscle. WT and EHD1^−/−^ endplates were singly innervated, even when the endplate was partitioned (Figure 6B). Because WT fibers also displayed partitioned endplates, it is unlikely that the partitioning represented an aberrant structure or resulted from the loss of function of EHD1.

### Eps15 homology domain-containing (EHD)1^−/−^ mouse skeletal muscle does not show pathology or a reduction in contractile force compared with wild-type skeletal muscle

**Figure 7:**
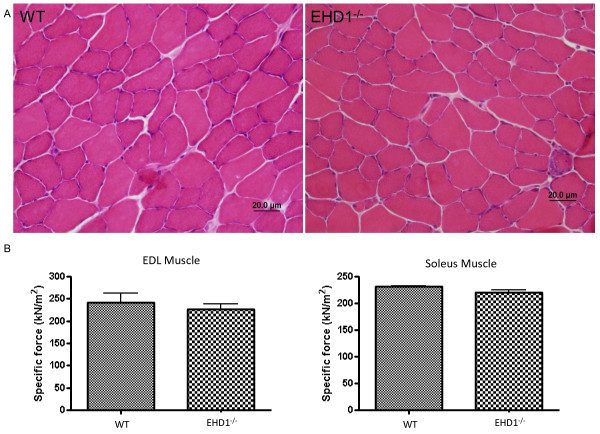
**Eps15 homology domain-containing (EHD)1**^**−/−**^**skeletal muscle shows normal pathology and generates average specific force.** Panel **(A)** Stainnig of tibialis anterior (TA) skeletal muscle cryosections (8 μm) from three WT and three EHD1^−/−^ mice did not show no degeneration or regeneration of fibers in EHD1^−/−^ mice, as shown by the absence of centralized nuclei. There was an absence of infiltrating white blood cells, also suggesting normal pathology (hematoxylin and eosin). **(B)** The force generated during a muscle contraction was measured *in vivo* in three WT and three EHD1^−/−^ EDL and soleus muscles each. EHD1^−/−^ EDL and soleus muscles showed no deficit in the amount of force generated during a muscle contraction (n = 3 per group).

Because EHD1^−/−^ skeletal muscle showed no overt synaptic defects, we assessed the overall muscle structure using hematoxylin and eosin (H&E) staining and assessed muscle function by measuring the intrinsic capacity to generate force during a muscle contraction. H&E-stained cross-sections of TA muscle (8 μm; n = 3 per group) were devoid of invading white blood cells, hypotropic and hypertrophic fibers, and regenerated fibers with centrally located nuclei (Figure 7A). Furthermore, there was no deficit in specific force generation in EHD1^−/−^compared with WT skeletal muscle. The specific force of the EDL (226 ± 23 kN/m^2^) and the soleus (220 ± 9 kN/m^2^) muscles from three EHD1^−/−^ mice showed no deficit compared withthe EDL (241 ± 37 kN/m^2^) and soleus (231 ± 2 kN/m^2^) muscle of the three WT mice (Figure 7B). All force measurements were normalized to muscle cross-sectional area. H&E staining of the EDL and soleus cross-sections had normal histology as previously described in the TA skeletal muscles, which is consistent with normal skeletal muscle function (data not shown). Indirectly, the maintenance of contractile force further supports healthy muscle structure and function in the absence of EHD1.

## Discussion

In this study, we characterized EHD1 and its family members through morphological mapping using *in vivo* recombinant gene expression in WT and EHD1^−/−^ mice. We found localization of EHD1 at the NMJ primary clefts of mouse TA skeletal muscle in addition to extrasynaptic regions of myofibers at lower levels. Furthermore, EHD1^−/−^ muscle fibers had normal NMJ morphology, histology, and function despite the deficient mice having a lower body mass and size. Muscle-wide expression of EHD1 was previously shown [30] by regional transcriptome profiling of laser capture microdissected synaptic and non-synaptic areas of rat TA skeletal muscle.

Transmission electron microscopy studies show an abundance of vacuoles in the space between secondary clefts at the NMJ [31], presumably to assist in receptor-mediated endocytosis and receptor recycling at the membrane. It seems that EHD family members localize to these vacuoles; based on the localization profiles in this study, EHD1, EHD3, and EHD4 are more likely than EHD2 to localize to these structures. Future studies using ultrastructural analyses and immunolocalization of EHD proteins should help to address this prediction. A functional role of EHD1 at the NMJ would suggest that loss of EHD1 should result in decreased receptor recycling at the membrane, forcing the formation of alternate endplates from newly synthesized receptors and the loss of proteins that interact with the extracellular matrix. The lack of morphological changes in EHD1^−/−^ endplates is therefore striking. Based on our earlier work with EHD4 in ear and testis and EHD3 in renal glomerular endothelial cells [32-34], the lack of an apparent phenotypic abnormality associated with lack of EHD1 at the NMJ could be explained by compensation by other paralogs, most likely EHD3 and EHD4, given their high similarity in protein sequence, domain structure, and localization. Our quantitative analyses of EHD4 expression in EHD1^−/−^ versus WT mice support this view (Figure 4). The loss of RME-1 (EHD1) in *Caenorhabtidis elegans* results in the accumulation of large vesicles in the intestines, suggesting a trafficking defect [4]. This defect was rescued by the expression of each one of the human EHD proteins, suggesting a conserved and redundant role of these paralogs. EHD3 has the greatest sequence similarity and overlaps in function with EHD1 during endosomal transport, and double knockdown of EHD1 and EHD4 in HeLa cells resulted in aberrant distribution of endocytic protein complexes [35]. Furthermore, EHD3 directly interacts with ankyrin B (ankyrin 2) [18]. Ankyrin-2 L and G are both localized to the troughs of synaptic folds, and NMJ morphology is altered in ankyrin-2 L^−/−^ skeletal muscle [36,37]. We hypothesize that double-knockout mouse models of EHD1 with either EHD3 or 4 may result in aberrant NMJ morphology.

NMJ morphology may not be indicative of function, for several reasons. First, the level of AChRs at the endplate exceeds the minimum concentration required to reach the action potential threshold. Second, the development of primary cleft arborization occurs within the first postnatal week, which is well after the motor neuron makes contact with the muscle fiber [38]. However, arborized endplates are not a requirement to invoke an action potential, as frog endplates are completely linear. It will be interesting to determine if endplates of double-knockout mouse models show aberrant morphology, and if the skeletal muscle shows sign of muscle weakness, which would indicate impaired synaptic transmission and support our hypothesis that the lack of observed defects in EHD1^−/−^ skeletal muscle is the result of compensation by other members. If this proves to be true, these double-mutant mice may be useful to further study receptor recycling and turnover, and to test the ‘signaling endosome’ hypothesis of tyrosine kinase receptors at the NMJ.

## Conclusions

In this study, we characterized the localization and function of a previously unknown NMJ protein. EHD1 is a NMJ protein that localized to the primary synaptic cleft. EHD1^−/−^ mouse skeletal muscle shows no functional deficit or endplate structural defects. Furthermore, this study provides experimental support for the hypothesis that paralogs of this family are compensatory in function. EHD4 also localized to the primary synaptic cleft, and was increased in EHD1^−/−^ mouse skeletal muscle, suggesting a compensatory function for this family member.

## Competing interests

The authors declare they have no competing interests.

## Authors’ contributions

SM performed all methods except for the specific force measurements, and drafted and edited the manuscript. JHV performed the specific force measurements and wrote the corresponding sections of the manuscript. PA and SB performed the animal husbandry and breeding, coordinated the shipment of EHD1^−/−^ mice, and established the correct reactivity of the lots of peptide-specific antibodies against EHD1-4 used in the present studies. HB made the EHD1^−/−^ mice available for these studies, shared his expertise on the function of EHD paralogs and their relationship to improve the design of experiments with antibodies and mice, and edited the manuscript. EPH assisted in the experimental design and manuscript editing. All authors read and approved the final manuscript.

## Authors’ information

SEM is a predoctoral student in the Biochemistry and Molecular Biology Program of the Institute for Biomedical Sciences at the George Washington University. This work is from a dissertation to be presented to the above program in partial fulfillment of the requirements for a PhD degree.
